# Are depression and anxiety associated with disease activity in rheumatoid arthritis? A prospective study

**DOI:** 10.1186/s12891-016-1011-1

**Published:** 2016-04-11

**Authors:** Faith Matcham, Sheila Ali, Katherine Irving, Matthew Hotopf, Trudie Chalder

**Affiliations:** Department of Psychological Medicine, Institute of Psychiatry, Psychology and Neuroscience, King’s College London, 10 Cutcombe road, London, SE5 9RJ UK; South London and the Maudsley NHS Foundation Trust, London, UK; King’s College Hospital NHS Foundation Trust, London, UK

**Keywords:** Depression, Anxiety, Rheumatoid arthritis, Prospective, Disease activity

## Abstract

**Background:**

This study aimed to investigate the impact of depression and anxiety scores on disease activity at 1-year follow-up in people with Rheumatoid Arthritis (RA).

**Methods:**

The Hospital Anxiety Depression Scale (HADS) was used to measure depression and anxiety in a cross-section of RA patients. The primary outcome of interest was disease activity (DAS28), measured one-year after baseline assessment. Secondary outcomes were: tender joint count, swollen joint count, erythrocyte sedimentation rate and patient global assessment, also measured one-year after baseline assessment. We also examined the impact of baseline depression and anxiety on odds of reaching clinical remission at 1-year follow-up.

**Results:**

In total, 56 RA patients were eligible for inclusion in this analysis. Before adjusting for key demographic and disease variables, increased baseline depression and anxiety were associated with increased disease activity at one-year follow-up, although this was not sustained after adjusting for baseline disease activity. There was a strong association between depression and anxiety and the subjective components of the DAS28 at 12-month follow-up: tender joint count and patient global assessment. After adjusting for age, gender, disease duration and baseline tender joint count and patient global assessment respectively, higher levels of depression and anxiety at baseline were associated with increased tender joint count and patient global assessment scores at 1-year follow-up.

**Conclusions:**

Symptoms of depression and anxiety have implications for disease activity, as measured via the DAS28, primarily due to their influence on tender joints and patient global assessment. These findings have implications for treatment decision-making as inflated DAS28 despite well controlled inflammatory disease markers may indicate significant psychological morbidity and related non-inflammatory pain, rather than true disease activity.

## Background

Rheumatoid Arthritis (RA) is a chronic autoimmune disease, with a worldwide adult prevalence of 0.2–1.2 % [1]. The disease is painful and progressive, leading to increasing levels of disability and systemic complications [2]. There is currently no cure for RA: treatment aims are to reduce pain and inflammation, delay joint erosion and maintain function [3]. Depression and anxiety are highly prevalent in rheumatoid arthritis (RA). According to a recent meta-analysis [4], 14.8 % of patients screen positive for depression when measured with the Hospital Anxiety and Depression Scale (HADS [5]) with a threshold of 11 or more indicating the presence of depression. This prevalence estimate is substantially higher than the 5.0 % level reported in the general population [6]. Symptoms of anxiety are also frequently reported in RA, with 25.1 % of RA outpatients screening positive for anxiety according to the 7-item Generalised Anxiety Disorder (GAD-7 [7]) questionnaire [8]. Anxiety and depression are sometimes grouped together and referred to as “common mental disorders”. Anxiety and depression may also be seen as symptoms which are present with varying degrees of severity in the population. In this paper, we refer to depression and anxiety scores as continuous indicators of symptom severity, rather than as disorders reaching a threshold of severity. This method is particularly beneficial as it maximises statistical power, and allows examination of the linear associations between mental health and disease outcomes [9, 10].

RA has significant implications for patient quality-of-life [11] and increased psychological symptoms in RA is associated with poorer patient outcomes including increased pain [12], fatigue [13], service usage [14] and increased risk of premature mortality [15]. The relationship between mental and physical health is bidirectional. Experiencing psychological distress may inflate the subjective severity of patient-reported symptoms such as pain and tenderness [16]. Additionally, psychological distress may impact health outcomes by influencing health behaviours such as medication adherence [17] and smoking [18]. Reduced levels of physical activity can result in de-conditioning, loss of natural endorphins and increased pain [19]. Furthermore, common mental disorders are associated with immune dysregulation [20–22].

A recent systematic review has found that only seven studies have examined the longitudinal relationships between depression and RA outcomes [23]. This review concluded that depression may worsen pain and disease activity, and reduce treatment efficacy, although evidence is limited by a small number of studies, often of poor quality. Studies frequently lacked a priori hypotheses, used convenience samples, and had inadequate adjustment for confounders [23]. We previously reported that increased psychological distress predicts increased disease activity (measured via the 28-joint Disease Activity Score; DAS28 [24]) and reduced odds of reaching clinical remission over a 2-year follow-up period, however this research is limited by its sub-standard identification of psychological distress, and its use of clinical trial data representing a relatively homogenous group of patients [25].

More research is needed to resolve these issues and replicate previous research findings, using robust methods of identifying depression and anxiety in a naturalistic patient sample. This could have substantial implications for the treatment of RA: if depression and anxiety are drivers of the non-inflammatory components of DAS28 disease activity, optimal RA treatment plans could involve the pharmacological or psychological management of depression and anxiety alongside RA. We therefore aimed to explore the relationship between depression and anxiety and disease activity after one year. We tested three hypotheses: 1) that increased depression and anxiety symptom severity at baseline would be significantly associated with increased DAS28 at 1-year follow-up; 2) that the relationship between depression and anxiety and DAS28 would be primarily driven by an association between depression and anxiety and the subjective DAS28 components: tender joint count (TJC) and patient global assessment (PGA); and 3) that increased levels of depression and anxiety at baseline would be associated with reduced odds of reaching clinical remission at 1-year follow-up.

## Methods

### Procedure and participants

**Fig. 1:**
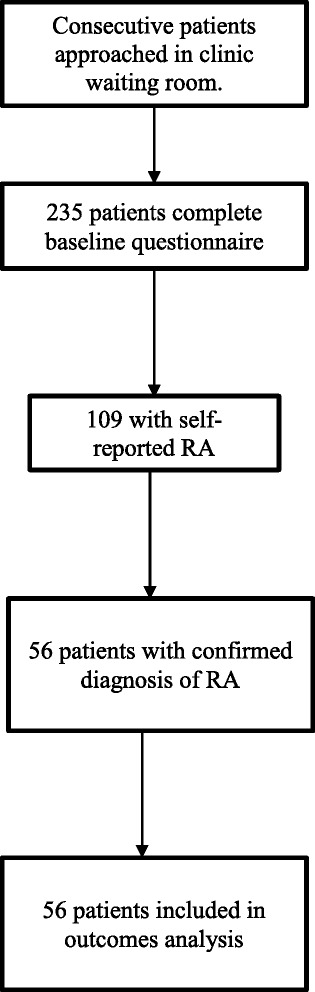
Flowchart of participant recruitment

This was a one-year prospective study, aiming to examine the psychological factors associated with disease outcomes in RA. Patients attending a rheumatology outpatient appointment at King’s College Hospital, London, were approached consecutively in the clinic waiting room and invited to participate. All patients were approached and consenting patients completed the questionnaires regardless of diagnosis. RA diagnosis was confirmed through checking their hospital records, to verify clinician-diagnosed RA. Only data from patients with RA diagnosis according to 2010 ACR/EULAR criteria [26] were included in the current analysis. Figure 1 shows the flow of participants through the recruitment and analysis strategy. The following inclusion criteria were required for the subsample described in this study: clinician confirmed RA diagnosis; aged 18 years or over; attending the hospital outpatient clinic. Patients were excluded if they had insufficient English to be able to read and understand the questionnaires.

Participants gave informed consent and were asked to complete a baseline questionnaire, which was completed during their hospital visit. Outcome data were obtained from patient medical records from appointments at 1-year (±3 months) after the baseline questionnaire was completed.

The study protocol was approved by the South East London Research Ethics Committee (REC reference number: 10/H0808/135).

### Data collection

#### Baseline/predictor variables

Baseline depression and anxiety were measured via the questionnaire administered in the hospital waiting room. The questionnaire packs included several measures of psychological, social, disease and work-related variables, however only responses to the HADS were included in the current study. The HADS consists of 14 items, 7 for symptoms of depression and 7 for anxiety symptoms. Each item has 4 possible interval response options¸ providing a score from 0–3 and the summed total gives a score out of 21 for each subscale, with higher scores indicating higher levels of depression or anxiety. The HADS depression and anxiety subscales have good discriminant validity when compared to psychiatric assessment [5], are internally consistent (α = 0.85 and 0.78 respectively), and have good convergent validity in RA patients [9, 10].

#### Follow-up/outcome variables

The primary outcome to test hypothesis 1 was DAS28. The DAS28 is considered to be the gold-standard indicator of disease activity: it is recommended by all major RA guidelines; is widely used both in clinical trials and as part of clinical practice; and takes into account subjective and objective indicators of disease severity [27]. Its subcomponents TJC, SJC, PGA and ESR were used as secondary outcomes, examining the impact of depression and anxiety on the subjective (TJC, PGA) and objective components (SJC, ESR) of disease activity (hypothesis 2). PGA is measured on a visual analogue scale, providing a patient perspective on their health status; scores range between 0 (worst health) and 100 (best health). Due to the composite nature of the DAS28, total scores can be inflated by non-inflammatory pain, demonstrated in high levels of PGA and TJC, despite well-managed inflammation [28]. We hope to investigate this by examining the overall DAS28 alongside individual assessment of its subcomponents.

To examine the impact of depression and anxiety on odds of reaching clinical remission after 1 year (hypothesis 3), the number of patients scoring <2.6 on the DAS28 at 1-year follow-up was established [29].

Only data from one follow-up appointment were included, with all disease outcomes identified from hospital records to match as closely as possible to 1 year post-baseline, with a ± 3-month window of acceptability.

### Statistical analysis

#### Missing data

Of the 56 eligible participants, one patient had missing baseline HADS data, and one had missing baseline DAS28 data. Six patients had missing DAS28 scores at follow-up: two did not attend a follow-up appointment within the given ± 3 month time-frame and four did not have their DAS28 measured within the ± 3 month time-frame.

Patients with missing outcome data did not significantly differ from patients without missing data in relation to their age, gender, baseline disease activity, ESR, TJC, PGA, SJC, depression, or anxiety (further details available on application from the author). This assessment revealed no systematic differences between participants with and without missing data and missing data were managed via multiple imputation. In addition to outcome data, baseline data, demographics and all covariates were used to impute the missing values, using chained equations with 13 cycles. The 13 datasets were separately analysed and combined using Rubin’s rules [30].

#### Hypothesis testing

ESR found to be non-normal in distribution and was log transformed prior to analysis. No other parametric assumptions were violated and therefore multiple linear regression models were created to test hypotheses 1 and 2. These models were created using Stata (version 11.2), providing unstandardised *b* coefficients, standard errors (SE), 95 % confidence intervals (CI) and *p*-values. Unadjusted and adjusted models were created. Adjustment included: disease duration, age, and baseline score of each outcome variable (for example, baseline DAS28 was adjusted for in the model predicting follow-up DAS28; baseline TJC was adjusted for in the model predicting follow-up TJC). Age was adjusted for due to the large age-range represented in the sample (26–83 years). Disease duration was selected as a covariate due to its strong association with outcome variables, established through preliminary bivariate correlation analyses. Baseline scores for outcome variables were adjusted for in order to ascertain the association between depression and anxiety irrespective of baseline severity.

To assess hypothesis 3, a logistic regression model was created to examine the association between baseline depression and anxiety scores and odds of reaching clinical remission at 1-year. An unadjusted model and one adjusted for disease duration, age, and baseline disease activity are presented.

## Results

### Participant characteristics

Figure 1 shows the flow of participants through the study. Of the 235 patients who completed the questionnaire, 109 (46 %) had self-reported RA. Of these, only 56 (51.3 %) had clinically verified RA. Table 1 shows the descriptive statistics of the 56 participants included in the current analysis. In total, 78.6 % of participants were female, and the mean age was 53.6 years. The majority of participants identified themselves as White (64.8 %). The mean time between baseline and follow-up measurements was 1.1 years (SD = 0.4).

**Table 1:** Baseline demographic, disease and psychological variables

Variable		N (%)	M(SD)	Observed range
Total N		56		
*Demographics*				
Female, N (%)		44 (78.6)		
Mean age			53.6 (13.4)	26–83
Ethnicity, N (%)	White	35 (64.8)		
	Asian	7 (13.0)		
	Black	11 (20.4)		
	Mixed	1 (1.9)		
	Not reported	2 (3.8)		
*Disease characteristics (baseline)*				
Mean disease duration (Years)			10.3 (9.2)	0.2–38
Experiencing comorbidity, N (%)		32 (57.1)		
Treatment type, N (%)	DMARD	38 (67.9)		
	Biologics	14 (25.0 %)		
	Not reported/other	4 (7.1 %)		
Rheumatoid factor positive N (%)		40 (71.4)		
Median ESR (IQR)			27.0 (12.0–44.0)	2–102
Mean TJC			5.2 (6.1)	0–24
Mean SJC			3.0 (2.9)	0–10
Mean PGA			45.8 (25.0)	1–90
Mean DAS-28			4.5 (2.2)	0.9–14.1
Clinical remission (DAS-28 < 2.6), N (%)		8.0 (14.6)		
*Psychological status (baseline)*				
Mean depression			7.4 (4.7)	0–20
Mean anxiety			7.7 (5.0)	0–20
*Disease characteristics (follow-up)*				
ESR, median (IQR)			22.5 (8.0–44.0)	2–114
TJC, M (SD)			4.0 (5.8)	0–28
SJC, M (SD)			3.2 (3.8)	0–16
PGA, M (SD)			44.6 (26.1)	1–100
DAS-28, M (SD)			4.7 (2.8)	0.5–17.1
Clinical remission (DAS-28 < 2.6), N (%)		8.0 (16.0)		

*M* mean, *SD* standard deviation, *DMARD* disease modifying anti-rheumatic drug, *NSAID* non-steroidal anti-inflammatory drug, *ESR* erythrocyte sedimentation rate, *TJC* tender joint count, *SJC* swollen joint count, *PGA* patient global assessment, *DAS-28* 28 joint disease activity scale, *HADS-D* hospital anxiety and depression scale depression subscale, *HADS-A* hospital anxiety and depression scale anxiety subscale

### Hypothesis 1: the relationship between baseline depression and anxiety and follow-up disease activity

Table 2 shows the results of Pearson’s correlation analyses examining bivariate associations between demographics, baseline variables and DAS28 at follow-up.

**Table 2:** Pearson correlational relationships between continuous demographic, baseline and follow-up variables

		Demographics			Baseline variables							Follow-up variables				
		Age	Disease duration	N. comorbidities	ESR	TJC	SJC	PGA	DAS-28	Depression	Anxiety	ESR	TJC	SJC	PGA	DAS-28
	Age	-														
	Disease duration	0.29*	-													
	Number of comorbidities	0.32	0.22	-												
Baseline Variables	ESR	0.10	0.27*	0.07	-											
	TJC	−0.13	0.07	0.13	−0.13	-										
	SJC	−0.14	−0.03	0.03	0.11	0.54**	-									
	PGA	−0.20	0.16	0.25	0.18	0.64***	0.45***	-								
	DAS-28	−0.17	0.13	0.03	0.44***	0.41**	0.47***	0.60***	-							
	Depression	−0.26	0.12	−0.16	0.04	0.25	0.23	0.44**	0.40**	-						
	Anxiety	−0.29*	−0.02	−0.11	0.03	0.16	0.19	0.31*	0.29*	0.76***	-					
Follow-up Variables	ESR	0.10	0.33*	−0.01	0.86***	−0.13	−0.02	0.16	0.37**	0.03	0.03	-				
	TJC	0.18	0.23	0.15	−0.18	0.24	0.00	0.32*	0.07	0.34*	0.23	0.02	-			
	SJC	0.33*	0.32*	0.16	0.23	0.01	0.01	0.11	0.07	0.16	0.08	0.44**	0.64***	-		
	PGA	0.19	0.33*	0.03	0.11	0.29*	0.19	0.46***	0.45***	0.45***	0.43**	0.22	0.37**	0.23**	-	
	DAS-28	−0.03	0.21	0.06	0.36**	0.07	0.08	0.35*	0.80***	0.39**	0.33*	0.47**	0.33*	0.36*	0.61***	-

*ESR* erythrocyte sedimentation rate, *TJC* tender joint count, *SJC* swollen joint count, *PGA* patient global assessment, *DAS-28* 28 joint disease activity scale, *HADS-D* hospital anxiety and depression scale depression subscale, *HADS-A* hospital anxiety and depression scale anxiety subscale. * *p* < 0.05, **p < 0.01, ***p < 0.001

Table 3 shows the results of multiple regression models, examining the impact of baseline depression and anxiety level on DAS28. The results of this analysis revealed that both depression and anxiety scores at baseline were associated with increased DAS28 at 1-year follow-up, however this relationship became non-significant after adjustment for covariates.

**Table 3:** Multiple regression model of unstandardized (b) coefficients for primary and secondary outcomes, measured 1-year follow-up, by depression/anxiety severity at baseline

	Primary outcome			Secondary outcomes											
	DAS28			ESR			SJC			TJC			PGA		
	b(SE)	95 % CI	p	b(SE)	95 % CI	p	b(SE)	95 % CI	p	b(SE)	95 % CI	p	b(SE)	95 % CI	p
*Unadjusted*															
Depression	**0.06 (0.02)**	**0.10,0.45**	**0.01**	0.01 (0.04)	−0.06,0.09	0.74	0.12 (0.13)	−0.14,0.38	0.35	**0.55 (0.22)**	**0.11,0.99**	**0.02**	**2.68 (0.80)**	**1.07,4.28**	**<0.01**
Anxiety	**0.04 (0.02)**	**−0.00,0.07**	**0.05**	0.02 (0.03)	−0.05,0.08	0.63	0.06 (0.11)	−0.16,0.29	0.57	0.34 (0.20)	−0.06,0.75	0.09	**2.30 (0.72)**	**0.85,3.75**	**<0.01**
*Adjusted* ^a^															
Depression	0.03 (0.02)	−0.00,0.06	0.07	0.01 (0.02)	−0.03,0.05	0.69	0.19 (0.13)	−0.07,0.45	0.16	**0.59 (0.23)**	**0.13,1.05**	**0.01**	**2.07 (0.80)**	**0.46,3.69**	**0.01**
Anxiety	0.02 (0.01)	−0.00,0.05	0.06	0.02 (0.02)	−0.02,0.05	0.37	0.15 (0.11)	−0.07,0.38	0.18	**0.44 (0.20)**	**0.04,0.85**	**0.03**	**2.13 (0.65)**	**0.81,3.45**	**<0.01**

*DAS28* 28-joint disease activity score, *ESR* erythrocyte sedimentation rate, *SJC* swollen joint count, *TJC* tender joint count, *PGA* patient global assessment

^a^Model adjusted for age, disease duration and baseline physical health for each variable (e.g. DAS28 at baseline adjusted for in DAS28 outcome assessment; ESR at baseline adjusted for in ESR outcome assessment; etc.)

Bold text denotes significant association

### Hypothesis 2: the relationship between baseline depression and anxiety and follow-up DAS28 components

Table 2 shows the results of Pearson’s correlation analyses examining bivariate associations between demographics, baseline variables and follow-up DAS28 components: ESR, TJC, SJC, and PGA.

Table 3 also shows the models created for the sub-components of the DAS28: ESR, SJC, TJC and PGA. These analyses revealed no significant associations between depression or anxiety and the more objective components: ESR and SJC. Coefficients were small, and did not reach a level of statistical significance. Increased depression was found to be significantly associated with increased follow-up TJC both before and after adjustment, with a one unit increase in HADS depression at baseline contributing to a 0.59 increase in TJC at follow-up. However the relationship between anxiety and TJC only reached statistical significance after adjusting for covariates, with an increase in HADS anxiety at baseline contributing to a 0.44 increase in TJC at follow-up. Similarly, depression was a significant predictor of PGA at 1-year follow-up both before and after adjustment: after adjustment, a one unit increase in HADS depression at baseline was associated with an increase in PGA of 2.07 units. Increasing anxiety was associated with increased follow-up PGA both before and after adjusting for covariates, with a post-adjustment increase of one unit in baseline HADS anxiety associated with a 2.13 increase in PGA at follow-up.

### Hypothesis 3: the relationship between depression and anxiety and odds of reaching clinical remission

At baseline, 8 (16.0 %) of patients were in clinical remission (DAS28 < 2.6) and 8 (16.0 %) were in remission at follow-up. Of the 8 in remission at baseline, 6 were still in remission at follow-up; the remaining 2 patients in remission at follow-up were new cases. Table 4 shows the results of the logistic regression model assessing the associations between depression and anxiety scores and odds of reaching clinical remission.

**Table 4:** Logistic regression model of association between depression and anxiety and odds of reaching clinical remission at 1-year follow-up

	DAS28 remission		
	OR (SE)	95 % CI	*p*
*Unadjusted*			
Depression	−0.15 (0.11)	−0.37,0.07	0.18
Anxiety	−0.04 (0.08)	−0.20,0.12	0.63
*Adjusted*			
Depression	−0.14 (0.17)	−0.48,0.19	−0.48
Anxiety	−0.01 (0.14)	−0.28,0.26	0.94

*OR* odds ratio, *SE* standard error, *CI* confidence interval

Neither depression nor anxiety at baseline were significantly associated with the odds of being in clinical remission at one-year follow-up.

## Discussion

We have found evidence to support one of the three hypotheses: 1) depression and anxiety scores at baseline were not significant predictors of DAS28 disease activity at 1-year follow-up after full adjustment; 2) depression and anxiety were significantly associated with the subjective components of the DAS28: TJC and PGA; 3) depression and anxiety were not significantly associated with the odds of reaching clinical remission at 1-year follow-up after adjusting for covariates. These results support our previous findings from clinical trial data showing a longitudinal relationship between baseline depression/anxiety and follow-up tender joints, patient global assessment, however we failed to replicate our previous finding of an association between depression and anxiety and disease activity [25]. The current study uses a more robust, validated method of identifying depression and anxiety, in a more heterogeneous clinical sample, with longer and more variable disease duration. This strengthens the evidence indicating a prospective relationship between depression and anxiety and poorer subjective disease outcomes in RA [23]. Our failure to replicate the association between depression and anxiety and odds of reaching clinical remission may be due to a lack of statistical power to predict a binary outcome.

There are several possible explanations for the associations found between depression and anxiety and patient global assessment and tender joint counts. The negative cognitions often experienced in depression and anxiety [31] may contribute to how RA patients interpret and perceive their symptoms [32]. Psychological distress is associated with worsened health behaviours such as failure to take medications as prescribed [17], and increased smoking [18], or may contribute to reduced physical activity [19], which can also contribute to worsened disease outcomes. The association between depression and anxiety and the subjective DAS28 elements would suggest that depression/anxiety impact perceptions and behaviours, rather than immune dysregulation. Further examination of mediators in this relationship is warranted and potential targets for investigation are negative cognitions, behavioural activity and health behaviours.

Increased levels of tenderness and poor patient global assessments, despite well-managed inflammation, can inflate DAS28 scores thereby reducing the perceived efficacy of treatment [28]. There is substantial evidence to suggest that depression and anxiety can be effectively treated in physical conditions [33, 34]. Given the importance of the DAS28 in clinical decision making, the routine detection and management of depression and anxiety may be a further strategy to improve disease management [8], and doing so would align with National Institute of Health and Clinical Excellence guidelines [35].

### Limitations

This study has some limitations to consider. Firstly, the sample size is small, limiting statistical power and the scope to control for pertinent covariates. Our selectivity about covariates, whilst evidence-based, may have influenced results, and inclusion of several other variables such as comorbidities and ethnicity would have been preferable. Future research would benefit from recruiting patients with a clinician-verified RA diagnosis, rather than relying on self-reported diagnosis. We have shown that only 51.4 % of patients with a self-reported RA diagnosis in fact, have clinically verified RA. A further consideration was the lack of sociodemographic data available; low socioeconomic status (SES) patients are typically under-represented in research samples [36]. As low SES is associated with increased susceptibility to depression [37] and RA [38], our findings may not be generalizable to the general RA population, although it is important to note that KCH caters mostly for patients in South East London, which has a higher level of deprivation than the England average [39, 40]. Additionally, we were unable to invite patients who could not read/write English to complete the questionnaires, which may further limit the generalisability of our findings to the wider, non-English-speaking population.

Additionally, no information was collected during recruitment about patients who declined to participate. Therefore we were unable to determine participation rate, examine any demographic, physical, or psychological determinants of non-participation, or examine between-group differences in patients who consented to participate and those who did not. Future replication of this study should attempt to record details for all patients approached for recruitment.

A final limitation is the lack of data available regarding medication usage throughout the follow-up period, for either RA or mental disorder. DAS28 outcomes may be substantially driven by treatment intensity and modality, and treatment decision-making may be influenced by patient mental-state [41]. The addition of medication data may add valuable information to the understanding of these relationships. Furthermore, the inclusion of DAS28 data at only one follow-up point means that any variation in disease activity throughout the follow-up period, or as a result of treatment, cannot be assessed.

Future research may also benefit from taking into account change in depression and anxiety. There is some prospective evidence suggesting initial depression levels predict between 37 and 58 % of the variance in follow-up mood scores in RA [42]. With a larger sample size, it would be interesting to stratify our results by change in mood over time, to see if the relationship between mood and disease activity alters by mood trajectory.

## Conclusions

This study supports the findings from previous research indicating an association between depression and anxiety scores at baseline and worsened disease outcomes [23, 25]. These findings have several implications. Inflated DAS28 in the context of clinically well controlled disease may indicate significant psychological morbidity rather than true disease activity. Regardless of direction of causality, the consistent association between depression and anxiety and disease variables is strong, and depression and anxiety may act as easily identifiable and manageable “psycho-markers” of adverse disease outcome [43]. We recommend that depression and anxiety be measured in routine clinical practice and as part of randomised controlled trials for new treatments in RA.

Finally, research is required to determine whether effective treatment of depression and anxiety can improve rheumatological outcomes. Psychological interventions have been used to successfully improve disease outcomes in diabetes and coronary heart disease [44], and our results highlight the need to test a similar approach in rheumatoid arthritis.

### Ethics and consent to participate

Participants gave informed consent, and all study procedures and the study protocol was approved by the South East London Research Ethics Committee (REC reference number: 10/H0808/135).

### Consent to publish

Not applicable.

### Availability of data and materials

All supporting data can be provided upon request to the authors.

## Abbreviations

ACR: American College of rheumatology
DAS28: 28 joint disease activity score
DMARD: disease modifying anti-rheumatic drugs
ESR: erythrocyte sedimentation rate
GAD7: 7-item generalised anxiety disorder questionnaire
HADS: hospital anxiety and depression scale
OR: odds ratio
PGA: patient global assessment
RA: rheumatoid arthritis
SJC: swollen joint count
TJC: tender joint count
